# Mental health phenotypes of well-controlled HIV in Uganda

**DOI:** 10.3389/fpubh.2024.1407413

**Published:** 2025-01-28

**Authors:** Leah H. Rubin, Kyu Cho, Jacob Bolzenius, Julie Mannarino, Rebecca E. Easter, Raha M. Dastgheyb, Aggrey Anok, Stephen Tomusange, Deanna Saylor, Maria J. Wawer, Noeline Nakasujja, Gertrude Nakigozi, Robert Paul

**Affiliations:** ^1^Department of Neurology, Johns Hopkins University School of Medicine, Baltimore, MD, United States; ^2^Department of Psychiatry and Behavioral Sciences, Johns Hopkins University School of Medicine, Baltimore, MD, United States; ^3^Molecular and Comparative Pathobiology, Johns Hopkins University School of Medicine, Baltimore, MD, United States; ^4^Department of Epidemiology, Johns Hopkins Bloomberg School of Public Health, Baltimore, MD, United States; ^5^Missouri Institute of Mental Health, University of Missouri - St. Louis, St. Louis, MO, United States; ^6^Rakai Health Sciences Program, Kalisizo, Uganda; ^7^Department of Psychiatry, Makerere University, Kampala, Uganda

**Keywords:** mental health, global, Uganda, HIV, cognition, depression, anxiety, PTSD

## Abstract

**Introduction:**

The phenotypic expression of mental health (MH) conditions among people with HIV (PWH) in Uganda and worldwide are heterogeneous. Accordingly, there has been a shift toward identifying MH phenotypes using data-driven methods capable of identifying novel insights into mechanisms of divergent MH phenotypes among PWH. We leverage the analytic strengths of machine learning combined with inferential methods to identify novel MH phenotypes among PWH and the underlying explanatory features.

**Methods:**

A total of 277 PWH (46% female, median age = 44; 93% virally suppressed [<50copies/mL]) were included in the analyses. Participants completed the Patient Health Questionnaire (PHQ-9), Beck Anxiety Inventory (BAI), and the PTSD Checklist-Civilian (PCL-C). A clustering pipeline consisting of dimension reduction with UMAP followed by HBDScan was used to identify MH subtypes using total symptom scores. Inferential statistics compared select demographic (age, sex, education), viral load, and early life adversity between clusters.

**Results:**

We identified four MH phenotypes. Cluster 1 (*n* = 76; *PTSD phenotype*) endorsed clinically significant PTSD symptoms (average PCL-C total score > 33). Clusters 2 (*n* = 32; *anxiety phenotype*) and 3 (*n* = 130; *mixed anxiety/depression phenotype*) reported minimal PTSD symptoms, with modest BAI (Cluster 2) and PHQ-9 (Cluster 3) elevations. Cluster 4 (*n* = 39; *minimal symptom phenotype*) reported no clinical MH symptom elevations. Comparisons revealed higher rates of sexual abuse during childhood among the *PTSD phenotype* vs. the *minimal symptom phenotype* (*p* = 0.03).

**Discussion:**

We identified unique MH phenotypes among PWH and confirmed the importance of early life adversity as an early risk determinant for unfavorable MH among PWH in adulthood.

## 1 Introduction

Human immunodeficiency virus (HIV) and depressive disorders are highly prevalent, co-occurring conditions, that remain among the top 10 causes of disability among people living in the eastern, Sub-Saharan Africa country of Uganda (1). According to the 2020 Uganda Population-based HIV Impact Assessment, a nationwide survey to estimate the prevalence and incidence of HIV, approximately 1.3 million adults are living with HIV (2). The prevalence of current depressive disorders among Ugandan adults is reported to range between 14 and 21% (3). Notably, people with HIV (PWH) are disproportionately affected by depressive disorders (estimates of 21–28%) (4–6). Thus, there remains an urgent need to better understand and treat depressive disorders in PWH in this region of the world.

In the “Treat All era,” HIV studies in Uganda have primarily focused on depression as a unidimensional disease entity, and most commonly as an isolated mental health (MH) disorder in PWH. There is ample evidence from the field of psychiatry that there is considerable heterogeneity in the clinical presentation and course of depression (7, 8). However, this multidimensionality is rarely examined in HIV epidemiological studies (6) even though PWH with depressive disorders exhibit markedly different profiles of somatic (e.g., sleep, appetite) and non-somatic symptoms (e.g., anhedonia, feelings of sadness or loss) (9). For example, depressed PWH could lose or gain weight, sleep too much or too little, or experience psychomotor agitation or retardation, each of which likely have different underlying mechanisms and unique treatment considerations. Furthermore, depression often does not occur in isolation, with high comorbidity observed in the context of anxiety disorders (e.g., phobias, generalized anxiety disorder) and post-traumatic stress disorder (PTSD) (10). Data from the United States National Comorbidity Survey Replication indicates that 72.1% of individuals with a depressive disorder also meet criteria for at least one other MH disorder over a 12 month period, including 59.2% with anxiety disorders (10). This co-occurrence is also common among PWH living in sub-Saharan Africa (11).

While most studies have focused on diagnostic prevalence or total symptom burden, there has been a shift toward identifying MH phenotypes. To date, few studies have employed data-driven methods to identify and characterize MH phenotypes among PWH (12–18). Early efforts outside of neuroHIV have defined MH phenotypes based on clinical symptoms, an approach that has yielded novel insights into the neurobiological basis underlying the heterogeneity of depression as well as potential therapeutic targets. For example, one study using data-driven approaches identified and validated three depression phenotypes based on item level responses to self-report questionnaires (insomnia, affective, and atypical symptoms) (19). When considering treatment response, antidepressants were most effective for individuals with the affective phenotype compared to the other two groups.

In the context of HIV, a recent large-scale, multi-site study used latent class analysis to empirically identify MH phenotypes based on current symptoms of emotional distress and substance use as well as childhood trauma, which is known to predict MH disorders and substance use disorders in adulthood (20, 21). Recent studies using data driven methods have also provided new insights into the mechanism of divergent HIV disease outcomes. For example, Chan *et al*. used group-based trajectory analysis to identify three distinct longitudinal cognitive phenotypes among PWH who initiated ART during acute infection (22). Interestingly, more severe symptoms of depression at the start of ART was the only variable that differed between the groups, with more depression evident among those in the lowest performing cognitive group. In the same cohort of PWH, Paul et al. reported that item-level responses on traditional MH questionnaires at the time of HIV diagnosis and treatment onset predicted CD4/CD8 T-cell inversion after 144 weeks of suppressive ART (23).

Clinical characteristics of PWH in cohorts in the global south differ from cohorts in the global north. In the North American AIDS Cohort Collaboration on Research and Design (NA-ACCORD), which represents HIV care in the United States and Canada, the prevalence and multimorbidity of age-associated conditions, substance use, and polypharmacy is high and is forecasted to increase by 2030 in PWH (24, 25). In contrast, neuroHIV studies in Uganda indicate minimal medical comorbidities (e.g., obesity, diabetes, hypertension), no psychiatric medication use (e.g., antidepressants, anxiolytics), balanced proportions of females and males, a different HIV subtype distribution [primarily D (59%) and A (23%)], and a preponderance of heterosexual HIV transmission with virtually no injection drug use (9, 14, 26). Furthermore, the way in which individuals experience symptoms of mental distress is intimately bound to their cultural context. As such, MH phenotypes among PWH described in studies in western countries may not generalize to PWH in Uganda. Understanding the constellation of factors that explain unique MH phenotypes among PWH, including factors that are potentially modifiable vis-à-vis prevention and/or intervention, has the potential to inform the development and implementation of tailored therapeutic strategies capable of improving the MH of PWH in Uganda and other regions of the world.

In this study, we first aimed to identify MH phenotypes among PWH in Uganda. We focused on symptoms of depression, anxiety, and PTSD given the high prevalence of these MH conditions among PWH globally (6, 11, 27–29). Second, we aimed to understand the unique attributes of each phenotype by interrogating symptom (i.e., item) level data from each MH measure. Third, we aimed to identify and characterize the sociodemographic (including history of early life adversity), HIV disease indices and cognitive factors that correspond to specific MH phenotypes.

## 2 Methods

### 2.1 Participants

We evaluated 277 PWH at the Rakai Health Sciences Program (RHSP)-supported HIV clinics and the Rakai Community Cohort Study. This open, community-based cohort includes participants residing in 40 communities in rural Rakai District, Uganda. Eligible participants were PWH aged 18 or older at the time of enrollment. Additional exclusion criteria for the overall study included severe cognitive or psychiatric impairment precluding written informed consent (participants answered questions to demonstrate their ability to understand the nature of the study and their competency to provide informed consent), physical disability preventing travel to the RHSP clinic for study procedures, known central nervous system (CNS) opportunistic infections, or prior CNS disease. This study was reviewed and approved by the Western Institutional Review Board (IRB00209786), the Uganda Virus Research Institute Research Ethics Committee (GC/127/789), and the Uganda National Council for Science and Technology (HS634ES).

### 2.2 Study visit assessments

Consenting participants were administered a comprehensive assessment battery that required approximately 5 h to complete. In brief, the battery consisted of a structured questionnaire to record sociodemographic characteristics, substance use, medical history, ART and non-ART medication use, MH and cognitive assessments, functional status assessments, and a neuromedical exam. HIV status was confirmed by rapid test, and CD4 cell count and plasma viral load were assessed.

#### 2.2.1 MH, cognitive, and motor assessments

Participants completed the Patient Health Questionnaire-9 (PHQ-9) (30) to assess depressive symptoms, the Beck Anxiety Inventory (BAI) (31) to assess anxiety symptoms, the PTSD Checklist–Civilian Version (PCL-C) to determine PTSD symptoms (32), and the sexual and physical abuse subscales on the Childhood Trauma Questionnaire to determine early life adversity (33). Translation and back-translation between English and Luganda were performed for each questionnaire. Prior studies demonstrate that PHQ-9 has high sensitivity and specificity in PWH in Uganda (34, 35). The CTQ has also been validated within adults in northern Uganda (36). While the PCL-C has not been validated in Uganda or other East African sample; the PCL-5 which is an updated version of the PCL-C for the DSM-V was validated in college students in Rwanda (37). For cognition and motor function, participants completed tests of psychomotor speed (Color Trails 1, Symbol Digit Modalities Test), cognitive flexibility (Color Trails 2), fine motor speed and dexterity (Grooved Pegboard), verbal learning and memory (WHO-UCLA Auditory Verbal Learning Test [AVLT]), and gross motor function (Timed Gait) that had been previously translated into Luganda and successfully employed in our prior studies (14, 38). Raw test scores were used in subsequent analyses. Research nurses administered and scored the tests after completing a thorough training and certification program (14).

#### 2.2.2 Neurological evaluation and functional assessments

The neuro evaluation included a structured questionnaire of neurological symptoms employed in our prior studies (14, 38) and a neurologic exam to document extrapyramidal signs, gait, strength, reflexes, and neuropathy signs (39). Karnofsky Performance Status (40) was used to measure functional status.

### 2.3 Statistical analyses

The analytic approach involved several steps. First, hierarchical density-based spatial clustering of applications with noise (HBDScan) (41) after dimension reduction with the UMAP algorithm (42) was implemented to identify MH phenotypes using total scores from the BAI, PHQ-9 and PCL-C. HDBScan is a hierarchical, density-based clustering method that utilizes a proximal distance to the nearest neighbor approach. In contrast to common clustering methods (e.g., K-means), HDBScan does not require *a priori* determination of the expected number or shape of the clusters. Additionally, outliers are defined as a unique cluster rather than forced integration into an otherwise homogeneous cluster. The UMAP algorithm is a flexible non-linear dimension reduction method that estimates the topology of the data (including nonlinear interactions) to maintain the structure of complex data even at lower levels of dimensionality.

Second, we utilized inferential univariate statistical methods to determine if the clusters differed (from the referent Cluster) on a select number of variables informed by the results of prior research studies (demographics, viral load, and early life adversity). Early life adversity was examined as categorical (none or minimal, low to moderate, moderate to extreme). *T*-tests were employed for continuous variables and Chi-Square tests were used for categorical variables where applicable.

Third, we employed a machine learning approach to investigate a much larger array and dimensionality of variables (Table 1) that could help explain differences in the MH clusters identified in the first statistical step. Given the homogeneity of Clusters 1 (*PTSD phenotype*) and 4 (*minimal symptom phenotype*), we focused the classification analysis on these two subgroups. Specifically, we applied gradient boosted multivariate regression (GBM) (43, 44) a form of ensemble machine learning that yields similar classification accuracy to more computationally intensive methods, such as Super Learner (45), while minimizing the risk of overfitting (17, 46–51). CatBoost (43, 44) was utilized to build the classification model in Python. Feature selection was completed using an in-house program based on SciKit-learn (52) and PDPBox (53). Class membership was determined using a probability score based on the sigmoid function (1/(1 + e^(−x))), 0.5 decision boundary, and gradient descent to minimize prediction error. Highly correlated features (r > 0.65) were managed by selecting the feature with the highest maximal information coefficient (MIC) value. We examined two classification models, one that allowed two-way interactions and one that did not allow interactions among the features. Our prior studies have consistently revealed that inclusion of two-way interactions provides unique insights regarding potential mechanisms that underlie more complex clinical phenotypes (17, 23, 47–49).

**Table 1:** Input features for the gradient boosted machine learning (GBM) analyses.

Demographics	Age at time of most recent seizure	Grooved pegboard non-dominant time to completion
Sex	# of seizures inpast 12 months	Grooved pegboard non- dominant total # of drops
Age	Seizures with febrile illness	Color Trails 1 time to completion
Marital status	Anti-seizure medication use	Color Trails 1 # of prompts
Educational attainment (years)	Hypertension medication use	Color Trails 1 # sequence errors
Currently taking ART (yes/no)	Cholesterol medication use	Color Trails 1 # near misses
History of brain infection	ART duration (months)	Color Trails 2 time to completion
Medical history	History of stroke	Color Trails 2 # of prompts
History of diabetes	History of meningitis	Color Trails 2 # sequence errors
History of hypertension	Smoke cigarettes, tobacco, or pipe	Color Trails 2 # near misses
TB medicine use (Niazid; historical and current)	Illicit drug use in past 2 years (yes/no?)	Symbol Digit Modalities total correct
Dapsone use	ART adherence	Timed Gait average time to completion
Flagyl use	Karnofsky	Mental Health
Sensory Symptoms (tingling, burning, numbness in hands or feet)	Karnofsky score	Childhood Trauma Questionnaire (CTQ) – item level responses
Balance difficulty or unsteadiness	Cognitive/Motor testing	CES-D positive affect – item level responses
History of fit or seizure	WHO Verbal Learning & Memory: Trials I-V, # Correct	Hopkins Symptom Checklist-25 – item level responses for depression subscale
History of epilepsy or epileptic fits	Grooved pegboard dominant time to completion	
Age at time of first seizure	Grooved pegboard dominant # of drops	

Multiple steps were employed to reduce overfitting. First, as noted above we employed a classification method that is more robust to overfitting than other methods such as support vector machines. Second, we focused on parsimonious models. Specifically, the number of features in the final algorithms was determined by model saturation, at which point the inclusion of additional features did not improve model performance by more than 1 SD from the base model. Third, model performance was determined using the F1 score which is a more conservative approach to determine model performance in unbalanced designs compared to AUC. F1 is the harmonic mean of the precision (i.e., positive predictive value; PPV) and recall (i.e., sensitivity). The highest possible value is 1.0, indicating perfect PPV and sensitivity. Fourth, we employed five-fold cross validation repeated five times (total of 25 trials) and utilized the average F1 score as the final metric of model performance. Five-fold cross validation is recommended over higher fold options in cases where the sample sizes are restricted (54, 55).

## 3 Results

### 3.1 Demographic characteristics at enrollment

The sample was comprised of 277 PWH (127 males, 150 females). The median age of the participants was 44 [interquartile range (IQR) = 38–50], and the median years of educational attainment was 6 (IQR = 4–8). Most individuals (94%) had an undetectable viral load (<50 copies/mL), and the majority (81%) were on the ART regimen efavirenz+ lamivudine+ tenofovir. Overall, 15.2% of the sample had PCL-C scores ≥33 which is considered indicative of possible PTSD. Eight percent of the sample met criteria for mild anxiety on the BAI, 3% moderate anxiety, and 1.4% severe anxiety. For depressive symptoms, 12% met criteria for mild, 4% moderate, and 1.1% moderately severe depression. Overall, self-reported comorbidities were low (see Supplementary Figure S1).

### 3.2 MH phenotypes defined by the clustering analysis

**Figure 1:**
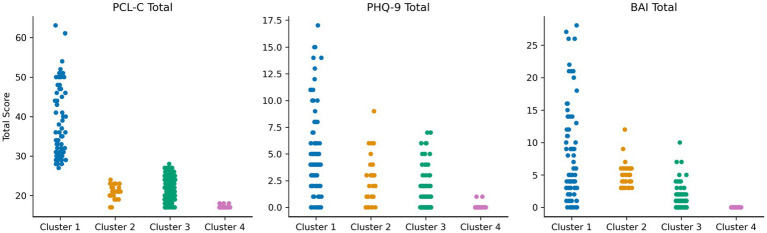
Scores on the PTSD checklist-civilian version (PCL-C), Patient health questionnaire-9 (PHQ-9), and Beck anxiety inventory (BAI) among the four mental health clusters in Figure. Strip plot demonstrating spread of scores on mental health inventories across the four clusters. Four mental health subtypes were identified. Cluster 1 (blue; PTSD phenotype) reported clinically elevated symptoms on the PCL-C, with a wide distribution of scores on the BAI and PHQ-9. Cluster 2 (orange; anxiety phenotype) reported lower PTSD symptoms, but depressive and anxiety symptoms in the mild–moderate range. Cluster 3 (green; mixed anxiety/depression phenotype) reported lower PTSD symptoms, with depressive and anxiety symptoms in the minimal-mild range. Cluster 4 (pink; minimal symptom phenotype) reported very low symptoms on all measures.

As depicted in Figure 1, the clustering algorithm identified four MH phenotypes. Cluster 1 (*N* = 76) included participants who endorsed a high frequency of PTSD symptoms on the PCL-C, with an average total score that was above the clinical threshold. Individuals in this cluster also reported symptoms on the BAI and the PHQ-9; however, the average total scores were below the clinical thresholds. Cluster 1 was designated as a *PTSD-phenotype*. Clusters 2 (*N* = 32) and 3 (*N* = 130) included individuals who reported minimal to mild levels of anxiety on the BAI and/or depression on the PHQ-9, with modestly higher scores on the BAI reported by individuals in Cluster 2 [mean (M) = 5.03, standard deviation (SD) = 1.91] compared to individuals in Cluster 3 (*M* = 1.15, SD = 1.54). As such, Cluster 2 was designated as an *anxiety phenotype* and Cluster 3 was designated as a *mixed anxiety/depression phenotype*. Cluster 4 (*n* = 39) was comprised of individuals who reported no clinical elevations on the BAI, PHQ-9, or the PCL-C. Cluster 4 was designated as a *minimal symptom phenotype*. Clustering analysis metrics indicated good cluster cohesiveness and robustness based on analysis of Silhouette score (see Supplementary Figures S2–S4).

Overall, Cluster 1 (*PTSD phenotype*) exhibited higher scores on the PCL-C, PHQ-9, BAI total scores (*p’s* < 0.001) compared to Cluster 4. Cluster 2 (*anxiety phenotype*) reported higher scores on the PCL-C, PHQ-9, and BAI total scores (*p*’s < 0.01) compared to Cluster 4. Finally, Cluster 3 (*mixed anxiety/depression phenotype*) exhibited higher PHQ-9 (*p* = 0.03) and PCL-C (*p* < 0.001) total scores compared to Cluster 4.

### 3.3 Item-level analysis of MH symptoms by cluster

**Figure 2:**
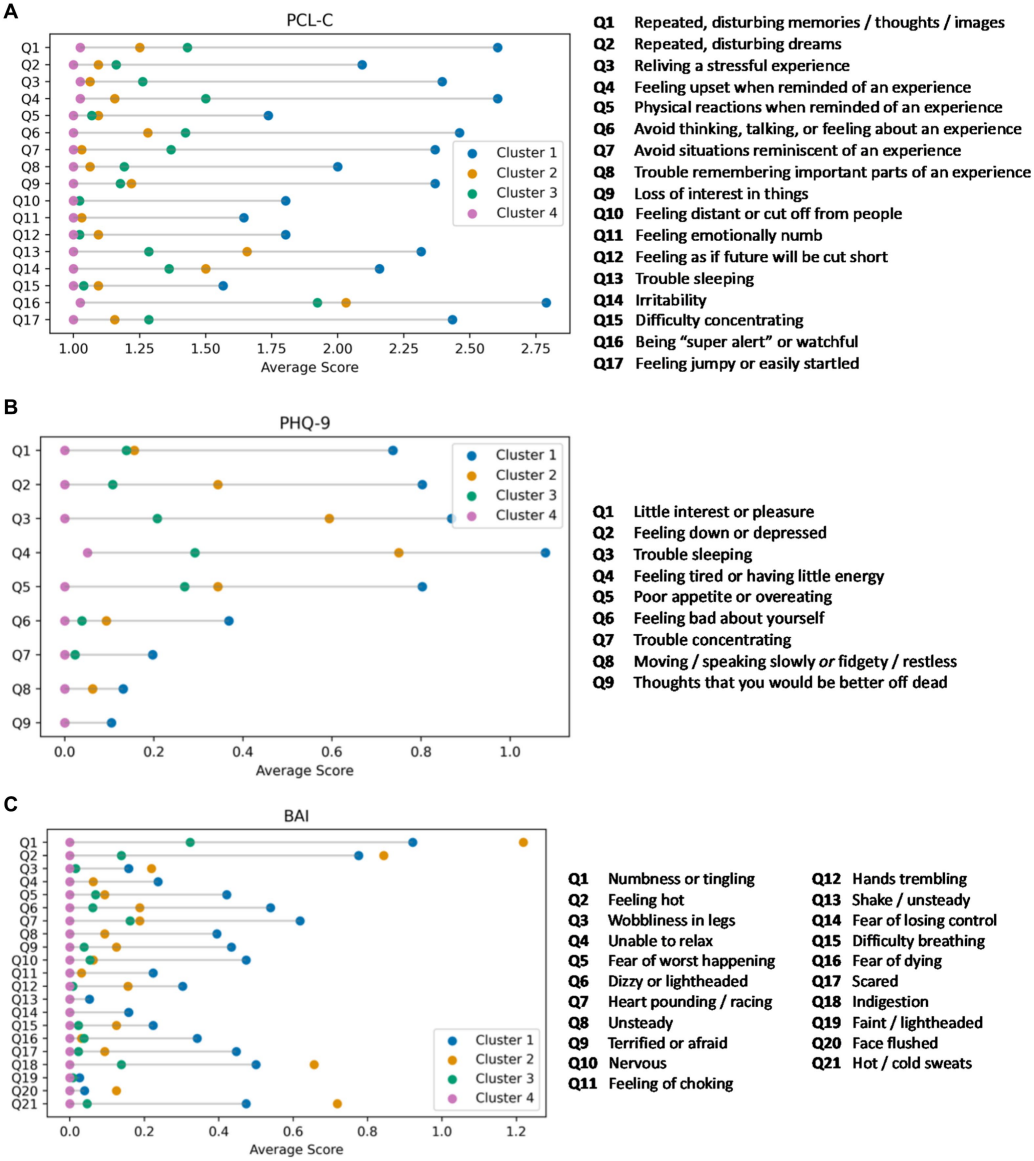
**(A)** Item level responses on the PTSD Checklist-Civilian version (PCL-C) by cluster. Lollipop plots displaying the mean scores on individual PCL-C items for each of the mental health clusters. Cluster 1 (blue; PTSD phenotype) reported clinically elevated PCL-C symptoms. Clusters 2 (orange; anxiety phenotype), 3 (green; mixed anxiety/depression phenotype), and 4 (pink; minimal symptom phenotype) reported lower scores in stepwise fashion on each item as well as the PCL-C total score. **(B)** Item level responses on the Patient Health Questionnaire-9 (PHQ-9) by cluster. Lollipop plots displaying the mean scores on the item level responses to the PHQ-9. Cluster 1 (blue; PTSD phenotype) reported, on average, clinically elevated PHQ-9 symptoms. Cluster 2 (orange; anxiety phenotype) reported somatic symptoms, while Clusters 3 (green; mixed anxiety/depression phenotype), and 4 (pink; minimal symptom phenotype) endorsed few symptoms. **(C)** Item level responses on the Beck Anxiety Inventory (BAI) by cluster. Lollipop plots displaying the mean scores on individual BAI items for each of the mental health clusters. Clusters 1 (blue; PTSD phenotype) and 2 (orange; anxiety phenotype) reported greater total BAI symptom burden, but with distinct profiles between the two groups (more affective symptoms for Cluster 1 and more physical symptoms for Cluster 2). Clusters 3 (green; mixed anxiety/depression phenotype) and 4 (pink; minimal symptom phenotype) had low anxiety symptoms on each BAI item and on the BAI total score.

As expected from the primary clustering results, individuals in Cluster 1 reported a high burden of symptoms on the PCL-C, consistent with a *PTSD phenotype* (Figure 2A). Of interest, cognitive symptoms on the PCL-C (e.g., difficulty concentrating) were infrequently endorsed by individuals in this cluster. It is of note that the average age of individuals in Cluster 2 is 49, nearly 5 years older than the average age of study participants in the other three clusters. On the PHQ-9, individuals in Cluster 1 endorsed more severe ratings of anhedonia (Figure 2B) compared to the other groups. Similar to the results of the BAI, individuals in Cluster 2 reported a high burden of vegetative symptoms of depression. Clusters 3 and 4 were similar in terms of item level responses on the PHQ-9. On the BAI, individuals in Cluster 1 reported more severe ratings than the other groups except for physical symptoms (e.g., “feeling hot”), which were more frequently reported by individuals in Cluster 2 (Figure 2C). Individuals in Cluster 2 reported a low rate of affective symptoms of anxiety (e.g., scared, fear of losing control), but a high rate of physical symptoms.

### 3.4 Inferential comparisons between MH phenotypes

Comparisons between cluster group 1, 2, and 3 and Cluster 4 (*minimal symptom phenotype*) were examined for differences in demographic, viral load, and early life adversity variables. Cluster 1 (*PTSD phenotype*) exhibited a higher rate of childhood sexual abuse (*p* = 0.03), but not physical abuse (*p* = 0.26) versus Cluster 4. Cluster 2 (*anxiety phenotype*) was older (*p* < 0.001) and reported lower rates of childhood sexual abuse (*p* = 0.04) versus Cluster 4. Cluster 3 (*mixed anxiety/depression phenotype*) did not differ from Cluster 4 on these factors. See Table 2 for full descriptive statistics per Cluster.

**Table 2:** Demographic and clinical characteristics for the total sample and by cluster.

	Total Sample (*n* = 277)	Cluster 1	Cluster 2	Cluster 3	Cluster 4
	M (SD)	(*n* = 76: 27%)	(*n* = 32: 12%)	(*n* = 130: 47%)	(*n* = 39: 14%)
		M (SD)	M (SD)	M (SD)	M (SD)
*Variables used in the cluster analysis*					
Mental health indices					
BAI total score	3.25 (5.12)	7.76 (7.46)	5.03 (1.91)	1.15 (1.54)	0 (0)
PHQ-9 total score	2.18 (3.17)	5.09 (4.19)	2.34 (2.22)	1.08 (1.60)	0.05 (0.22)
PCL-C total score	25.10 (9.11)	37.14 (8.80)	20.81 (1.67)	21.52 (3.1)	17.1 (0.31)
*Variables used in inferential statistical and machine learning analyses*					
Age	44.05 (8.77)	42.92 (9.54)	49.59 (8.08)	44.04 (8.49)	41.74 (6.86)
Male sex, *n* (%)	127 (46)	28 (37)	13 (41)	65 (50)	21 (54)
Years of education	6.21 (3.68)	6.00 (3.62)	6.47 (3.94)	6.03 (3.71)	6.97 (3.49)
Undetectable viral load, *n* (%)	259 (94)	67 (88)	30 (94)	124 (95)	38 (97)
Early life adversity-CTQ					
PA					
None or minimal	230 (83)	55 (72)	28 (88)	114 (88)	33 (85)
Low to moderate	26 (9)	10 (13)	3 (9)	9 (7)	4 (10)
Moderate to extreme	21 (8)	11 (15)	1 (3)	7 (5)	2 (5)
SA					
None or minimal	208 (75)	44 (58)	27 (84)	105 (81)	32 (82)
Low to moderate	33 (12)	11 (14)	5 (16)	15 (11)	2 (5)
Moderate to extreme	36 (13)	21 (28)	0 (0)	10 (8)	5 (13)
*Variables used in machine learning analyses only*					
Married, *n* (%)	162 (58)	37 (49)	13 (41)	89 (68)	23 (59)
CTQ-*item level scores*					
PA by family required medical	1.05 (0.35)	1.09 (0.50)	1.09 (0.53)	1.04 (0.23)	1.00 (0.00)
PA by family leaving marks	1.30 (0.76)	1.50 (0.90)	1.16 (0.45)	1.25 (0.74)	1.23 (0.67)
Punished with a hard object	1.26 (0.71)	1.32 (0.79)	1.22 (0.66)	1.22 (0.65)	1.31 (0.80)
Belief was PA	1.24 (0.66)	1.43 (0.85)	1.19 (0.54)	1.14 (0.51)	1.23 (0.67)
Others noticed PA	1.21 (0.65)	1.38 (0.85)	1.09 (0.39)	1.18 (0.59)	1.10 (0.50)
Any attempt at SA	1.20 (0.55)	1.45 (0.77)	1.00 (0.00)	1.09 (0.36)	1.23 (0.63)
Threatened if refused SA	1.13 (0.47)	1.20 (0.54)	1.03 (0.18)	1.08 (0.35)	1.23 (0.74)
Forced to do/watch sexual things	1.21 (0.62)	1.41 (0.82)	1.13 (0.49)	1.13 (0.46)	1.15 (0.67)
Molested	1.23 (0.64)	1.50 (0.95)	1.06 (0.25)	1.12 (0.41)	1.18 (0.56)
Belief was SA	1.20 (0.57)	1.38 (0.71)	1.06 (0.25)	1.14 (0.53)	1.15 (0.54)
Cognition					
WHO AVLT total learning	47.83 (8.05)	49.13 (8.10)	47.28 (8.10)	47.42 (8.32)	47.10 (6.97)
WHO AVLT delayed recall	10.00 (2.48)	10.42 (2.46)	9.56 (2.71)	10.02 (2.48)	9.46 (2.26)
WHO AVLT recognition	13.68 (1.83)	14.04 (1.25)	14.06 (1.13)	13.63 (1.64)	12.82 (3.17)
Pegs-dominant	81.40 (23.05)	83.95 (25.87)	88.26 (25.12)	79.60 (21.70)	77.00 (18.64)
Pegs-nondominant	94.28 (27.94)	97.84 (31.60)	105.50 (31.57)	89.34 (23.77)	94.32 (27.23)
Color Trails 1	90.32 (32.12)	92.59 (36.45)	99.90 (35.07)	89.33 (29.67)	81.57 (27.09)
Color Trails 2	188.37 (68.13)	196.33 (72.62)	197.86 (66.97)	186.40 (67.08)	172.19 (62.74)
Color Trails 1-near misses	0.16 (0.44)	0.19 (0.40)	0.06 (0.25)	0.13 (0.38)	0.26 (0.72)
Color Trails 2-near misses	0.31 (0.65)	0.30 (0.52)	0.48 (0.89)	0.30 (0.64)	0.18 (0.69)
Symbol digit	18.94 (10.24)	19.17 (10.60)	17.69 (9.62)	18.83 (10.17)	19.97 (10.59)
Timed gait	11.18 (1.62)	11.46 (1.85)	11.08 (1.28)	11.19 (1.55)	10.70 (1.55)
Medical history, *n* (%)					
Diabetes	2 (1)	1 (1)	0 (0)	1 (1)	0 (0)
Hypertension	14 (5)	7 (9)	1 (3)	6 (5)	0 (0)
Sensory Symptoms	82 (30)	38 (50)	15 (47)	27 (21)	2 (5)
Balance difficulty	6 (2)	4 (5)	2 (6)	0 (0)	0 (0)
Fit or seizure	4 (1)	3 (4)	0 (0)	1 (1)	0 (0)
Smoke	27 (10)	10 (13)	5 (16)	9 (7)	3 (8)
Medication use, *n* (%)					
Niazid	195 (70)	48 (63)	24 (75)	91 (70)	32 (82)
Dapsone	11 (4)	1 (1)	3 (9)	5 (4)	2 (5)
Flagyl	5 (2)	4 (5)	1 (3)	0 (0)	0 (0)
Antihypertensive	8 (3)	5 (7)	0 (0)	3 (2)	0 (0)

BAI, Beck Anxiety Inventory; CTQ, Childhood Trauma Questionaire; M, mean; PA, physical abuse; PCL-C, PTSD checklist-civilian version; PHQ-9, patient health questionnaire-9; SA, sexual abuse; SD, standard deviation; sensory symptoms include tingling, burning, numbness in hands or feet; WHO AVLT, World Health Organization Auditory Verbal Learning Test.

### 3.5 Machine learning classification of PTSD vs. minimal symptom phenotype

**Figure 3:**
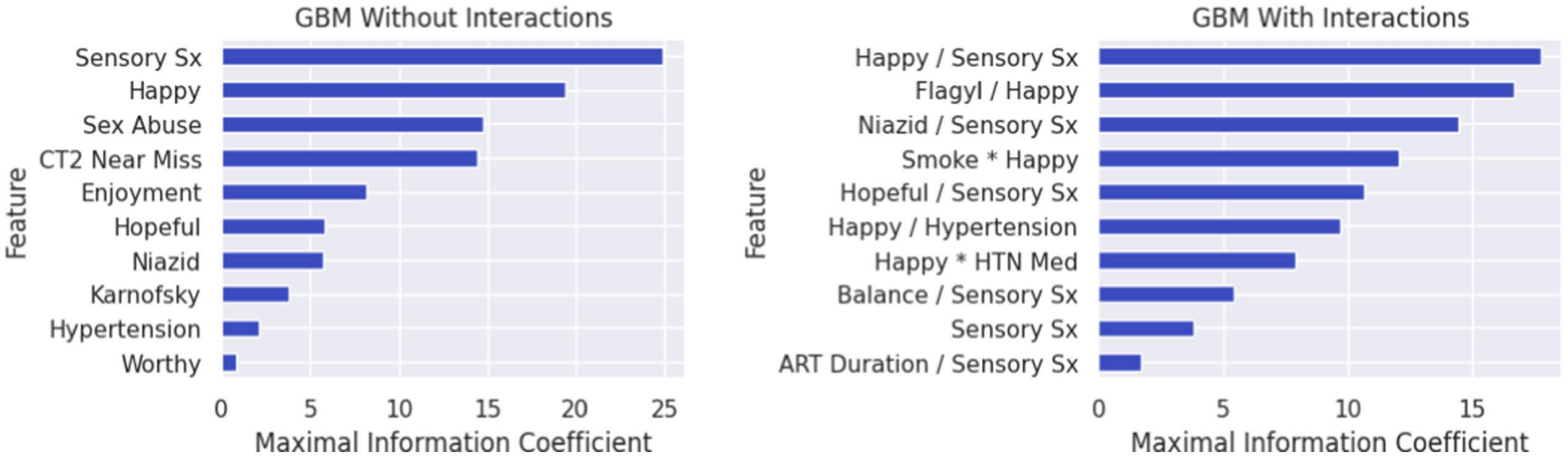
Feature lists depicting the rank order (highest to lowest) of variables classifying individuals in the PTSD phenotype (Cluster 1) vs. the minimal symptom phenotype (Cluster 4). In Figure Variables that collectively classified individuals into the PTSD phenotype (Cluster 1) versus the minimal symptom phenotype (Cluster 4) for the gradient boosted machine learning (GBM) without interaction features (left) and the GBM with interaction features (right). For the GBM with interaction features, multiplication between variables reflects synergies (risk linked to change in the same direction), whereas division between variables reflects divergence in the directionality. Features in rank order of relevance to classification without interactions include: **(Feature 1)** endorsing sensory symptoms (e.g., tingling, burning); **(Feature 2)** ratings of happiness over the past week; **(Feature 3)** belief that sexually abused in childhood; **(Feature 4)** near misses on Color Trails 2; **(Feature 5)** ratings of life enjoyment over the past week; **(Feature 6)** ratings of hopefulness over the past week; **(Feature 7)** taking Niazid; **(Feature 8)** Karnofsky score; **(Feature 9)** hypertension; **(Feature 10)** feelings of worthiness over the past week. The feature list for the model with interactions includes: **(Feature 1)** ratings of happiness over the past week and sensory symptoms (e.g., tingling, burning); **(Feature 2)** taking Flagyl and ratings of happiness over the past week; **(Feature 3)** taking Niazid and sensory symptoms (e.g., tingling, burning); **(Feature 4)** smoking and ratings of happiness over the past week; **(Feature 5)** ratings of hopefulness over the past week and sensory symptoms (e.g., tingling, burning); **(Feature 6)** ratings of happiness over the past week and hypertension; **(Feature 7)** ratings of happiness over the past week and taking anti-hypertensive medications; **(Feature 8)** balance difficulties and endorsing sensory symptoms (e.g., tingling, burning); **(Feature 9)** endorsing sensory symptoms (e.g., tingling, burning); **(Feature 10)** duration of ART and endorsing sensory symptoms (e.g., tingling, burning).

**Figure 4:**
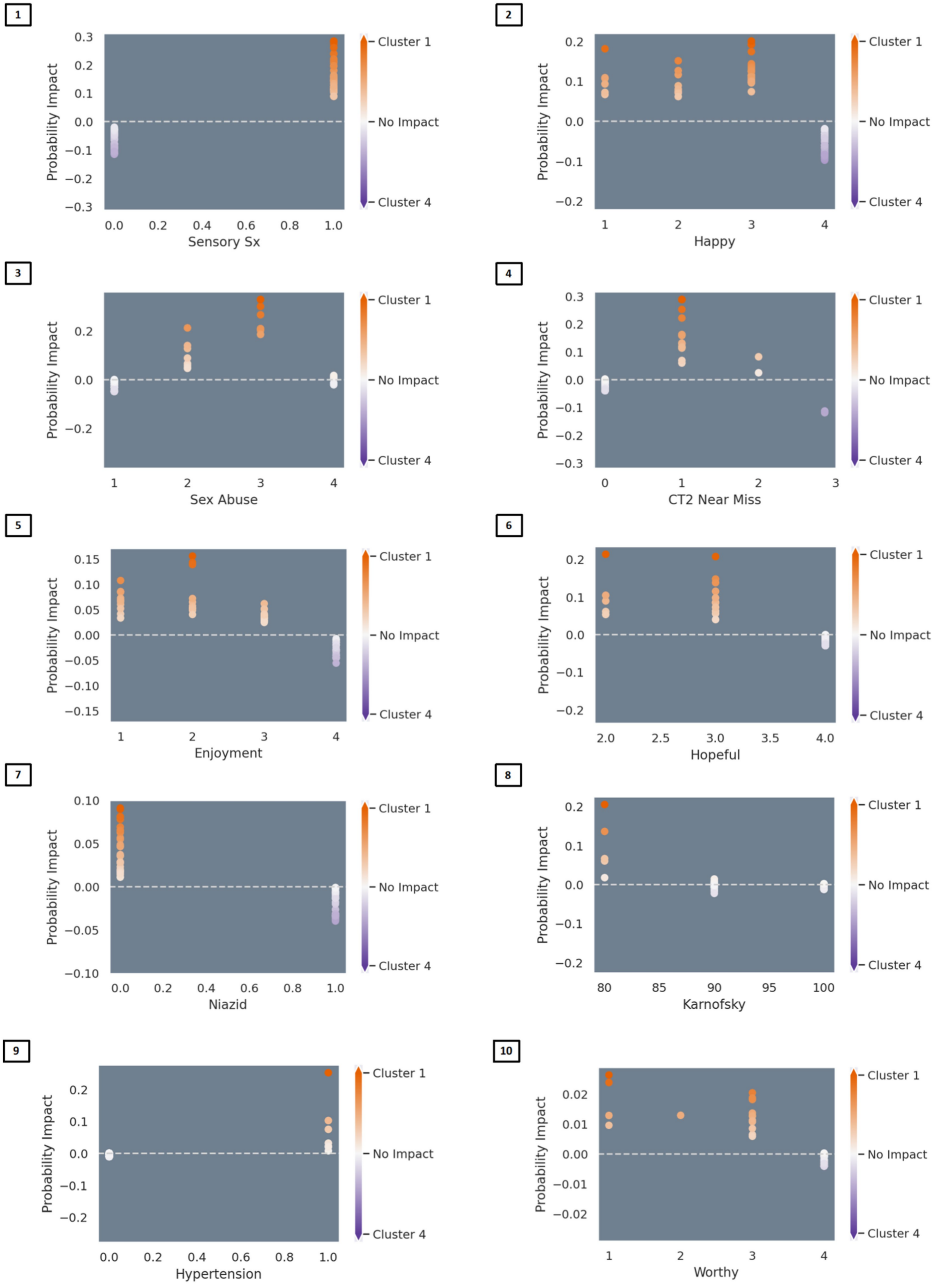
Partial dependency plots depicting the directionality of associations between variables and classification into the PTSD versus the minimal symptom phenotypes. In Figure Partial dependency plots depicting linear and nonlinear relationships between variables and classification into the PTSD versus the normative MH phenotypes. Red represents association with the PTSD phenotype (Cluster 1) and blue represents association with the normative phenotype (Cluster 4). Predictors of classification into the PTSD phenotype included: **(Feature 1)** endorsement of sensory symptoms (e.g., tingling, burning); **(Feature 2)** lower ratings of happiness over the past week; **(Feature 3)** belief that sexually abused in childhood; **(Feature 4)** 1–2 near misses on Color Trails 2; **(Feature 5)** lower ratings of enjoying life over the past week; **(Feature 6)** lower ratings of hopefulness over the past week; **(Feature 7)** not taking medications for tuberculosis (i.e., niazid); **(Feature 8)** lower Karnofsky score; **(Feature 9)** history of hypertension; **(Feature 10)** lower ratings of worthiness/greater feelings of worthlessness.

**Figure 5:**
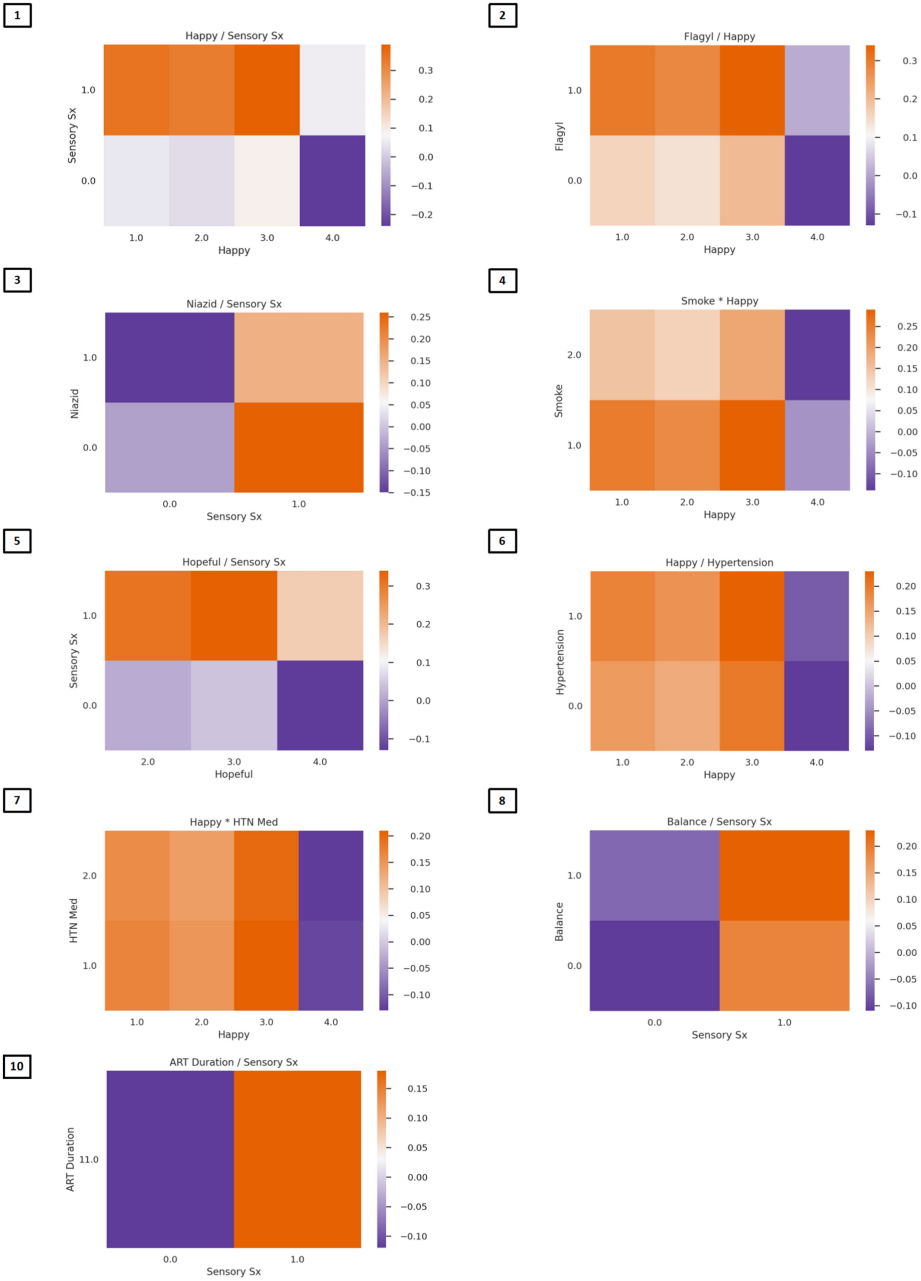
Heat maps depicting two-way associations between variables in the classification model. In Figure Heatmaps depict color-coded probabilities of classification in the PTSD phenotype (red) vs. the normative phenotype (blue). Predictors of PTSD phenotype membership are presented in descending order of feature importance. Multiplication between variables reflects synergies (risk linked to change in the same direction), whereas division between variables reflects divergence in the directionality. **(Feature 1)** Lower ratings of happiness and endorsement of sensory symptoms; **(Feature 2)** Lower ratings of happiness and taking Flagyl; **(Feature 3)** Endorsement of sensory symptoms and not taking Niazid; **(Feature 4)** Lower ratings of happiness and smoking history corresponded to classification in the PTSD phenotype group; **(Feature 5)** Lower ratings of hopefulness and endorsement of sensory symptoms; **(Feature 6)** lower ratings of happiness and history of hypertension; **(Feature 7)** lower ratings of happiness and taking hypertensive medications; **(Feature 8)** endorsement of both sensory symptoms and balance problems corresponded to classification in the PTSD phenotype; **(Feature 9)** endorsement of sensory symptoms (no heat map); **(Feature 10)** endorsement of sensory symptoms and lower duration of ART corresponded to classification in the PTSD phenotype.

The algorithm to classify individuals into Cluster 1 (*PTSD phenotype*) vs. Cluster 4 (*minimal symptom phenotype*) yielded an F1 score of 79% for the model without interactions (see Supplementary Table S1 for full model performance metrics). The classification algorithm was built from 10 features (Figure 3; Supplementary Table S2) including (1) tingling, burning, or numbness in the feet or hands; (2) response to CES-D item, “During the past week, I was happy.”; (3) response to CTQ item, “When I was growing up, someone tried to make me do sexual things or watch sexual things.”; (4) number of near misses on Color Trails 2; (5) response to CES-D item, “During the past week, I enjoyed life.”; (6) response to CES-D item, “During the past week, I felt hopeful about the future.”; (7) taking tuberculosis medication (i.e., niazid); (8) Karnofsky score; (9) history of hypertension; and (10) response to CES-D item, “During the past week, I felt I was just as good as other people.” The classification algorithm allowing for two-way interactions (see Figure 3; Supplementary Table S3) yielded an F1 score of 81% using the following 10 features: (1) happiness over the past week and tingling, burning, or numbness in the feet or hands; (2) use of Metronidazole and happiness over the past week; (3) use of tuberculosis medication and tingling, burning, or numbness in feet or hands; (4) smoking cigarettes, tobacco, or a pipe and happiness over the past week; (5) tingling, burning, or numbness in feet or hands and hopefulness over the past week; (6) hypertension and happiness over the past week; (7) hypertension medication and happiness over the past week; (8) tingling, burning, or numbness in feet or hands and balance difficulty or unsteadiness when walking; (9) tingling, burning, or numbness in feet or hands (a non-interactive feature); (10) time taking ART and tingling, burning, or numbness in feet or hands. Partial dependency plots in Figure 4 depict the directionality of each feature in relation to cluster classification (*PTSD phenotype* vs. *minimal symptom phenotype*). The heatmaps depicted in Figure 5 visualize the interactions as they relate to the classification results.

## 4 Discussion

Using novel analytic techniques, this study examined depression, anxiety, and PTSD symptoms within a sample of PWH in Uganda, with the aim of identifying distinct MH phenotypes. We identified four phenotypes: high PTSD symptoms, moderate anxiety, mixed anxiety/depression, and minimal clinical symptoms. Prior research suggests that internalizing disorders are highly heterogenous and that individuals can present with varied profiles of physical and psychological symptoms. For example, there are over 14,000 symptom combinations and over 200 ways that individuals can meet symptom criteria for major depressive disorder (MDD) (56, 57). Additionally, depression, anxiety, and PTSD are highly comorbid, with significant overlap in the diagnostic criteria of MDD, generalized anxiety disorder, and PTSD (58, 59). Our findings of four distinct MH phenotypes support prior work on the heterogeneity of internalizing disorders and highlights the importance of taking a multidimensional approach to understanding MH within HIV (Table 1).

Within the study, 86% of the sample fell within one of the clinical phenotypes, with the remaining cases defined as outliers. The most common phenotype was *mixed anxiety/depression*, which comprised 47% of the sample. The second most common group was the *PTSD phenotype* (27%), followed by the *minimal symptom phenotype* (14%), and the *anxiety phenotype* (12%). These results suggest that the norm for PWH is to experience MH symptoms, most commonly mild anxiety and depression, followed by high levels of PTSD symptoms.

In addition to cluster analyses, this study examined item-level responses to detect further distinctions in the MH phenotypes. The *PTSD phenotype* reported more physical symptoms, higher anhedonia, and fewer cognitive symptoms than the *anxiety phenotype*. Individuals in the *anxiety phenotype* reported fewer affective symptoms and more physical symptoms of anxiety. All four clusters endorsed histories of childhood physical and sexual abuse. However, childhood sexual abuse rates were higher in the PTSD phenotype.

Individuals in Clusters 2 and 3 (*anxiety* and *mixed anxiety/depression phenotypes*) reported similar levels of anxiety and depressive symptoms. However, upon inspection at the item level, the distinction between these groups becomes more apparent. Individuals in the *anxiety phenotype* endorsed somatic/physical BAI items at a higher rate than all other clusters. These items included experiencing numbness or tingling, feeling hot, indigestion, and hot or cold sweats. They were also more likely to endorse difficulty with sleeping or feeling tired on the PHQ-9. This finding suggests that while the total symptom burden may be similar between the *anxiety* and *mixed anxiety/depression* phenotypes, the nature of the symptoms is distinctive. It also highlights both the importance and benefits of conducting item-level analyses to discover the determinants of distinct MH phenotypes.

Additionally, our results are consistent with the well-documented finding that childhood trauma is a risk factor for MH disorders. Although individuals across all groups (including Cluster 4-*minimal symptom phenotype*) endorsed early life adversity, childhood abuse was only a significant risk factor for distinguishing Cluster 1 (*PTSD phenotype*) versus Cluster 4. Of note, the participants in Cluster 1 endorsed a high frequency of PTSD symptoms with an average total PCL-C score that was above the clinical threshold whereas Clusters 2 (*anxiety phenotype*) and 3 (*mixed anxiety/depression phenotype*) had total scores on the MH indices that were below the threshold. Thus, childhood abuse appears to be a risk factor only for PWH that report MH symptoms that are clinically significant. With respect to the *minimal symptom phenotype*, the higher endorsement of childhood sexual abuse suggests a level of resilience within some of the sample. Alternatively, this group may be underreporting their MH symptoms. Future research that examines factors that may predict whether PWH with histories of childhood sexual abuse experience elevated trauma symptoms as adults will be beneficial.

The average age of the moderate *anxiety phenotype* (49.59 years) was about 6–8 years older than the other phenotypes. Furthermore, the *anxiety phenotype* (59.4% female) comprised of 10–20% more women than the other clusters. When the item-level responses that distinguish the *anxiety phenotype* are examined within the context of these sociodemographic differences, it raises the possibility that these symptoms (numbness/tingling, feeling hot, indigestion, hot/cold sweats) may represent peri-menopausal features rather than anxiety. Thus, older women experiencing perimenopause may be potentially misclassified as falling within the anxiety group. Further examination of the symptom presentation of older women with HIV is therefore warranted.

To further understand differences between the *PTSD* and *minimal symptom phenotypes*, we utilized machine learning techniques to identify a combination of features that distinguished the two groups. Results from the models with and without interactions revealed that sensory symptoms (e.g., tingling, burning) and lower levels of happiness were prominent features associated with classification into the *PTSD phenotype*. Additionally, results from the models that allowed for up to two-way interactions revealed that use of Flagyl, Niazid, and hypertension medications in combination with sensory symptoms and lower ratings of happiness contributed to model performance. These findings emphasize the complexity of MH difficulties within the sample and the importance of understanding other components of health beyond those captured by traditional psychological measures in order to understand MH within the group.

Another important finding that warrants comment from the machine learning analysis is that the only cognitive feature that distinguished between the *PTSD* and *minimal symptom phenotypes* was the number of near misses on Color Trails 2. Typically, the only outcome examined on Color Trails is total completion time. Our finding suggests that the number of near misses should be considered as an additional cognitive outcome in neuroHIV studies.

Overall, results from this study highlight the frequent endorsement of somatic symptoms as a component of MH within a Ugandan sample. These findings are in line with prior research that suggests that individuals in Uganda are more likely to endorse somatic symptoms of MH (60–62). Similar findings have been reported in other low-income countries in the global south (63). This underscores the importance of taking a culturally sensitive and informed approach to MH assessment within this population.

This study had some limitations. First, we did not have a control group of people without HIV or those with other chronic disease who completed all three MH questionnaires. Without a comparison group, it is difficult to determine whether the observed MH phenotypes are unique to PWH or if similar patterns might be found in other populations, limiting the specificity of conclusions. Second, both the self-report measures and cognitive measures were designed using Western samples and therefore likely have cultural biases. Although many of these measures have been previously validated in Uganda or nearby countries, they likely still have some cultural bias such that some psychological and cognitive components of the Ugandan sample’s presentations may be misrepresented or missed during measurement. Future research that involves cultural adaptations or of, or addendums to, mental health measures would be beneficial to offer further insight and understanding of mental health symptoms and phenotypes within Uganda. Additionally, information about adulthood trauma and exposure to types of childhood trauma beyond physical and sexual abuse was not collected; thereby we could not examine how these factors may relate to current MH symptoms or phenotype placement.

This study offers several treatment implications. Firstly, individuals reported high levels of somatic symptoms (e.g., tingling). Therefore, treatment on somatic concerns may offer some amelioration of MH distress. Additionally, difficulties with sleep was frequently reported across the clinical phenotypes; thus, sleep intervention may be a beneficial area of care for PWH in Uganda. Interactions identified in our model such as “happiness over the past week” combined with “sensory symptoms” help to emphasize the multifactorial relationship between clinical disorder and symptom manifestation, with the goal of using these findings both to corroborate existing clinical impression and to better focus future research efforts geared toward enhancing precision medicine. Additional studies of the features with and without interactions are needed to further interrogate clinical relevance (e.g., predicting response to PTSD treatment) are needed.

The results of this analysis highlight several critical observations that are directly relevant to ongoing NIH initiatives aimed at identifying, characterizing, and predicting unique biotypes among PWH. Specifically, we demonstrate compelling proof of concept that use of advanced data driven analyses can delineate distinct and clinically relevant subgroups among a large sample of PWH who are receiving suppressive ART. Further, our results underscore the importance of combining exploratory (hypothesis generating) and confirmation (hypothesis testing) analytic strategies to accurately characterize and explain differences between the data-driven MH phenotypes. Specifically, the results describe the potential misclassification/misdiagnosis of anxiety-related symptoms among select subgroups of PWH (e.g., older females). Finally, the results identify the importance of early life adversity, particularly sexual abuse in childhood as an early risk determinant for PTSD symptomology in adulthood. Additionally, individuals in Cluster 4 also reported a history of sexual abuse; yet they reported no elevations in depression, anxiety, or PTSD symptoms, consistent with resilience or under-reporting. Follow-up analyses will further investigate the stability of these groups over time as well as to further characterize explanatory mechanisms of risk vs. resilience in terms of MH phenotypes. Incorporating neurobiological (including CD4/CD8 T cell count), genetic, and/or physiological metrics could yield additional insights into MH phenotype mechanisms and support the clustering results.

In conclusion, these results underscore the significant heterogeneity in MH profiles reported by PWH who have achieved viral suppression with sustained use of ART. The clusters identify distinct clinical profiles that differ markedly in nature and severity of mental health symptoms. Importantly, the different mental health profiles were not discernible at the group level. Our findings underscore the need to conduct deeper phenotyping of mental health symptoms to discern unique risk profiles nested within large clinical cohorts. Furthermore, whereas prior studies have prioritized assessment of depression among PWH, our findings indicate that anxiety and PTSD symptoms are also prevalent among virally suppressed PWH and merit clinical attention.

## Data Availability

The raw data supporting the conclusions of this article will be made available by the authors, without undue reservation.
