# Supplementary material for: Mental health phenotypes of well-controlled HIV in Uganda
Source: Front Public Health. 2025 Jan 28;12:1407413. doi: 10.3389/fpubh.2024.1407413 (PMC11810918; doi:10.3389/fpubh.2024.1407413)
Supplement: Supplementary file 1 [file Data_Sheet_1.docx]

Supplementary Material

**1 Supplementary Table 1**

Metrics of model performance for the classification of individuals in the PTSD vs. minimal symptom phenotypes

| Model+Feature Type | AUC (M) | AUC (SD) | Accuracy (M) | Accuracy (SD) | F1 (M) | F1 (SD) | Precision (M) | Precision (SD) | Recall (M) | Recall (SD) |
| --- | --- | --- | --- | --- | --- | --- | --- | --- | --- | --- |
| GBM with Interactive | 0.87 | 0.05 | 0.77 | 0.05 | 0.82 | 0.01 | 0.91 | 0.02 | 0.74 | 0.02 |
| GBM with Non-Interactive | 0.87 | 0.02 | 0.79 | 0.01 | 0.84 | 0.02 | 0.89 | 0.02 | 0.8 | 0.04 |
| Logistic regression with Interactive | 0.85 | 0.05 | 0.78 | 0.04 | 0.8 | 0.01 | 0.88 | 0.01 | 0.74 | 0.02 |
| Logistic regression with Non-Interactive | 0.81 | 0.07 | 0.79 | 0.05 | 0.81 | 0.01 | 0.87 | 0 | 0.75 | 0.02 |

Area Under the Curve (AUC); Gradient Boosted Multivariate Regression (GBM)

# 2 Supplementary Table 2

Response frequencies for each feature identified in the gradient boosted machine learning (GBM) without interactions.

| Feature |  | | Cluster 1 (n=76)  n (%) | Cluster 4 (n=39)  n (%) |
| --- | --- | --- | --- | --- |
| **1** | Endorsing sensory Symptoms | | 38 (50) | 2 (5) |
| **2** | During the past week, I was happy | Rarely or none of the time | 35 (46) | 37 (95) |
|  |  | Some or little of the time | 26 (34) | 1 (3) |
|  |  | Occasionally or a moderate amount of time | 9 (12) | 1 (3) |
|  |  | Most or all of the time | 6 (8) | 0 (0) |
| **3** | When I was growing up, I believe that I was sexually abused | Never True | 56 (74) | 35 (90) |
|  |  | Rarely True | 12 (16) | 3 (8) |
|  |  | Sometimes True | 7 (9) | 1 (3) |
|  |  | Often True | 1 (1) | 0 (0) |
|  |  | Very Often True | 0 (0) | 0 (0) |
| **4** | Near misses on Color Trails 2 | 0 | 53 (70) | 35 (90) |
|  |  | 1 | 18 (24) | 1 (3) |
|  |  | 2 | 2 (3) | 0 (0) |
|  |  | 3 | 0 (0) | 2 (5.1) |
| **5** | During the past week, I enjoyed life | Rarely or none of the time | 37 (49) | 35 (90) |
|  |  | Some or little of the time | 20 (26) | 3 (8) |
|  |  | Occasionally or a moderate amount of time | 9 (12) | 1 (3) |
|  |  | Most or all of the time | 10 (13) | 0 (0) |
| **6** | During the past week, I was hopeful about the future | Rarely or none of the time | 59 (78) | 39 (100) |
|  |  | Some or little of the time | 12 (16) | 0 (0) |
|  |  | Occasionally or a moderate amount of time | 5 (7) | 0 (0) |
|  |  | Most or all of the time | 0 (0) | 0 (0) |
| **7** | Taking Niazid | | 48 (63) | 32 (82) |
| **8** | Karnofsky Score | 70 | 1 (1) | 0 (0) |
|  |  | 80 | 4 (5) | 0 (0) |
|  |  | 90 | 17 (22) | 6 (15) |
|  |  | 100 | 54 (71) | 33 (85) |
| **9** | Hypertension | | 7 (9) | 0 (0) |
| **10** | During the past week, I felt I was just as good as other people | Rarely or none of the time | 60 (79) | 39 (100) |
|  |  | Some or little of the time | 11 (14) | 0 (0) |
|  |  | Occasionally or a moderate amount of time | 1 (1) | 0 (0) |
|  |  | Most or all of the time | 4 (5) | 0 (0) |

# 3 Supplementary Table 3

Computed values for each feature identified in the gradient boosted machine learning (GBM) with interactions.

| Feature | Cluster 1 (n=76) | Cluster 4 (n=39) |
| --- | --- | --- |
| 1 | During the past week, I was happy most or all of the time / No sensory symptoms  **(n=20, 26%)** | During the past week, I was happy most or all of the time / No sensory symptoms **(n=35, 90%)** |
| 2 | Not taking Flagyl /  During the past week, I was happy most or all of the time **(n=32, 42%)** | Not taking Flagyl /  During the past week, I was happy most or all of the time **(n=37, 95%)** |
| 3 | Taking Niazid /  Yes sensory symptoms **(n=25, 33%)** | Taking Niazid /  No sensory symptoms  **(n=31, 79%)** |
| 4 | Does not smoke cigarettes, tobacco, or a pipe *  During the past week, I was happy most or all of the time **(n=29, 38%)** | Does not smoke cigarettes, tobacco, or a pipe *  During the past week, I was happy most or all of the time **(n=34, 87%)** |
| 5 | During the past week, I was hopeful most or all of the time/ No sensory symptoms  **(n=30, 39%)** | During the past week, I was hopeful most or all of the time / No sensory symptoms  **(n=37, 95%)** |
| 6 | During the past week, I was happy most or all of the time / No history of hypertension **(n=33, 43%)** | During the past week, I was happy most or all of the time / No history of hypertension **(n=37, 95%)** |
| 7 | During the past week, I was happy most or all of the time * Not taking anti-hypertensive medication **(n=34, 45%)** | During the past week, I was happy most or all of the time * Not taking anti-hypertensive medication **(n=37, 95%)** |
| 8 | No balance difficulty or unsteadiness when walking /  Yes sensory symptoms  **(n=38, 50%)** | No balance difficulty or unsteadiness when walking /  No sensory symptoms  **(n=37, 95%)** |
| 9 | Yes sensory symptoms **(n=38, 50%)** | No sensory symptoms **(n=37, 95%)** |
| 10 | ART Duration **(n=11)** /  Yes sensory symptoms  **(n=1, 1%)** | Not applicable |

For the GBM with interaction features, multiplication between variables reflects synergies (risk linked to change in the same direction), whereas division between variables reflects divergence in the directionality. Sensory symptoms=tingling, burning, or numbness.

**4 Supplementary Figure 1**

Upset plot based on self-reported comorbidities in the cohort.


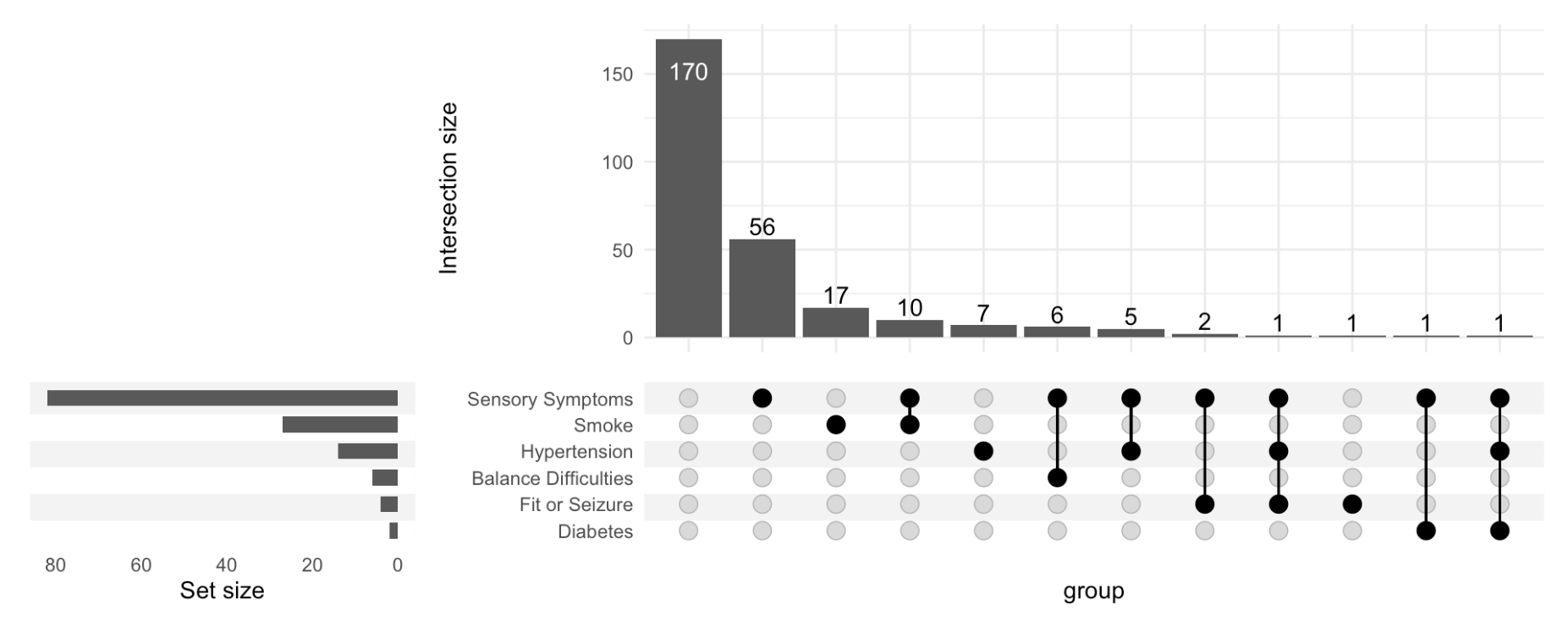


**
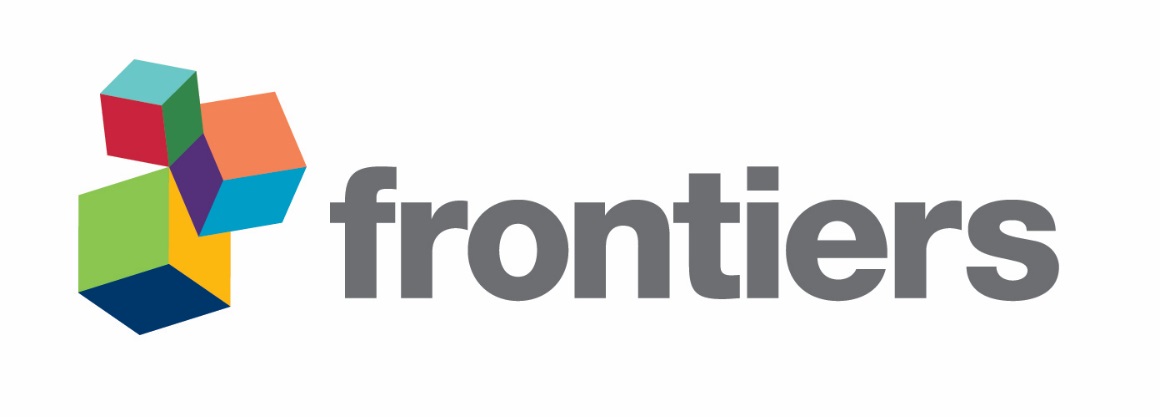
**

**5 Supplementary Figure 2**

Silhouette score is plotted in comparison to minimum cluster size


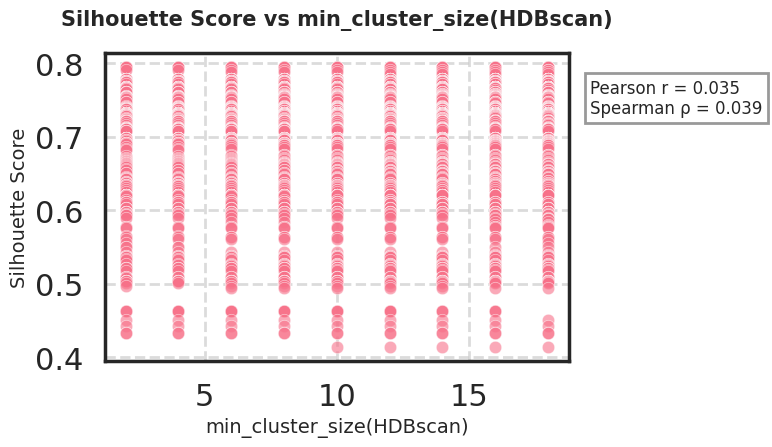


The Silhouette score, a measure of cluster cohesiveness, is plotted against the minimum cluster size hyperparameter that is optimized during clustering. A near-zero correlation between the Silhouette score and minimum cluster size suggests that changes to this parameter have little to no effect on the Silhouette score, indicating cluster quality that remains consistent regardless of cluster size.

**6 Supplementary Figure 3**

Silhouette score is plotted in comparison to minimum samples per cluster


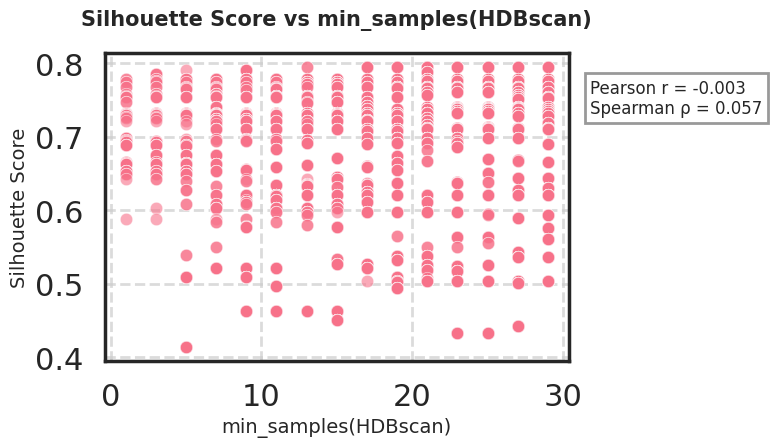


The Silhouette score, a measure of cluster cohesiveness, is plotted against the minimum number of samples hyperparameter that is optimized during clustering. A near-zero correlation between the Silhouette score and minimum number of samples suggests that changes to this parameter have little to no effect on the Silhouette score, indicating robustness of clusters regardless of number within each cluster.

**7 Supplemental Figure 4**

UMAP embeddings and HDBScan cluster assignments in 2D space
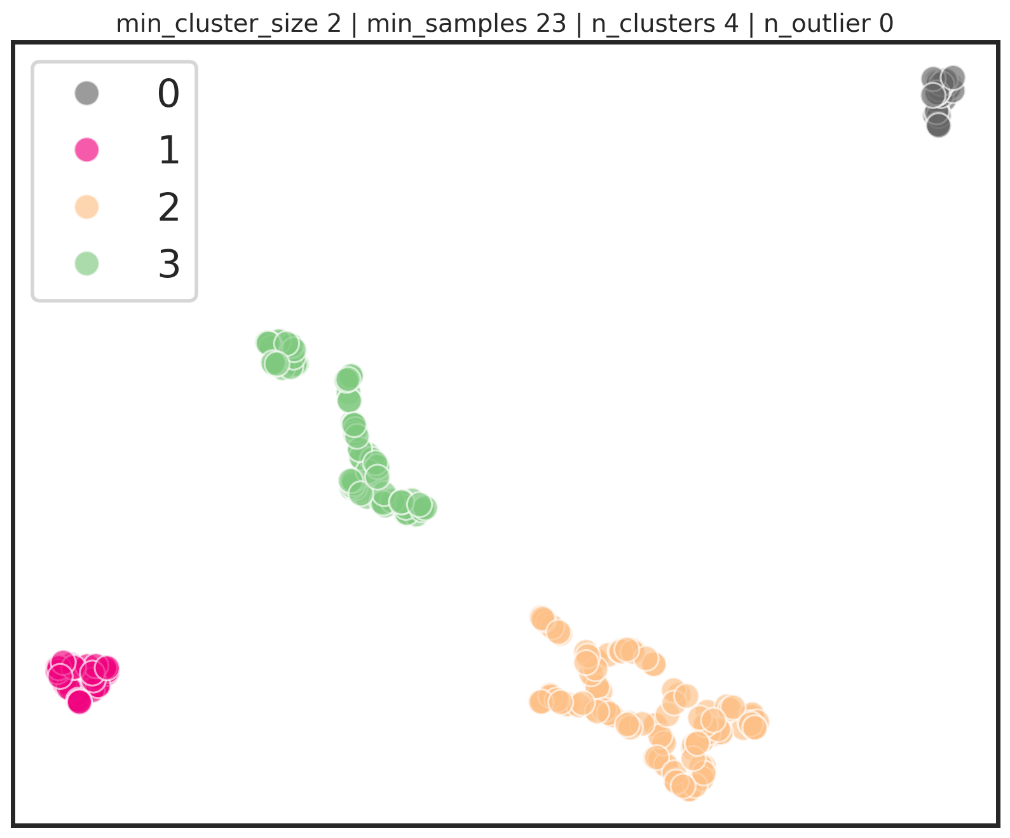


The spatial relationships between clusters in the final model are depicted in two-dimensional space along with associated hyperparameter settings. The cluster solution depicts good cohesiveness and separation of cluster groupings in a low-dimensional space.
